# An Online Community Intervention for Older Persons with Pre-Frailty and Frailty: Pilot Studies

**DOI:** 10.1093/geroni/igab046.3463

**Published:** 2021-12-17

**Authors:** Oleg Zaslavsky, Frances Chu, Shaoqing Ge, Andrew Teng, Shih-Yin Lin, George Demiris, Annie Chen

**Affiliations:** 1 Unversity of Washington, Seattle, Washington, United States; 2 University of Washington, University of Washington, Washington, United States; 3 University of Washington School of Nursing, Seattle, Washington, United States; 4 New York University, New York, New York, United States; 5 School of Nursing, University of Pennsylvania, University of Pennsylvania, Pennsylvania, United States

## Abstract

Online community interventions can support self-management in older populations but have rarely targeted symptomology of pre-frailty and frailty. To support older adults’ pre-frailty/frailty symptom management, we iteratively refined an approach entitled Virtual Online Community for Aging Life Experience (VOCALE) in three consecutive pilot studies (2018-2020). These studies employed asynchronous online discussions in which participants were asked to respond to weekly prompts. A study facilitator moderated the discussion, encouraging participants to respond to both the prompts and comments of other participants. In the first pilot (n=8), participants engaged in a collective exploration of different symptoms of pre-frailty and frailty. The second (n=10) and third (n=10) pilots employed a hybrid approach including collaborative exploration and learning of different problem-solving therapy skills over eight weeks. The mean age of participants of the three pilots combined was 80.6 (SD = 7.0). Most participants were female (71%). Participant attrition ranged from 20-25%. Many participants who completed the study noted that they enjoyed the discussions. The participants also found the moderators' follow-up questions and support timely and engaging. Additionally, we observed small but positive changes in self-efficacy measures. These pilot studies have confirmed that older adults with pre-frailty and frailty are interested, and can successfully engage in online community interventions, with the technical support and moderation provided, even during the initial stages of the COVID-19 pandemic, when lockdown policies were rolled out. Participation in the intervention was also associated with increased awareness of the need to be proactive in self-management concerning frailty-related symptoms.

